# Revision surgeries for tumor endoprostheses around the knee joint: a mid-long-term follow-up of 20 cases

**DOI:** 10.1186/s12957-022-02542-0

**Published:** 2022-03-10

**Authors:** Pengfei Zan, Hongsheng Wang, Zhengdong Cai, Jiakang Shen, Wei Sun

**Affiliations:** Department of Orthopedic Surgery, Shanghai General Hospital, Shanghai Jiao Tong University School of Medicine, No. 100 Haining Road, Hongkou District, Shanghai, China

**Keywords:** Tumor endoprostheses, Revision, Prosthetic fracture, Aseptic loosening, Infection

## Abstract

**Background:**

Tumor endoprostheses of the knee joint after limb salvage surgery is associated with high rates of complications, which has introduced great challenges to a delayed revision surgery. The aim of the study was to summarize the failures, functional outcomes and prosthetic survival in revision tumor endoprostheses of the knee joint.

**Methods:**

The clinical data of 20 patients with malignant tumors who received prosthetic revisions after limb salvage surgery from January, 2000 until January, 2018 were retrospectively reviewed. The cohort was constituted of 11 male and 9 female patients with a mean age of 34.1 years (range, 16 to 66 years). Infection cases received two-stage revisions after removing prostheses initially, while all other cases received one-stage revisions. Revision reasons and complications were well documented and analyzed.

**Results:**

All patients received complete follow-up with a mean time of 64.7 months (range, 27 to 155 months). A total of 6 (6/20, 30.0%) patients experienced a second complication after revision surgery, of whom, one patient with deep infection experienced repeated infections after prosthetic revision and received amputation surgery; one patient revised of prosthetic fracture experienced an infection and received a second-stage infection revision; one case revised of prosthetic loosening had deep infection receiving anti-infective therapy with prostheses still in position; one case having wound complication healed after receiving two times of debridement surgery; one MBGCT patient experienced a second aseptic loosening 6 years after the initial loosening thus undergoing a second revision; a recurrent osteosarcoma patient died of pulmonary metastasis 3 years after revision surgery. Kaplan-Meier survival curve indicated a 5-year survival rate of initial prostheses was 75%. The Musculoskeletal Tumor Society (MSTS-93) score [20.9 (range, 15 to 27 scores)] at 1 year after revision surgeries was significantly improved (*p* < 0.001) when compared with the score [17.2 (range, 13 to 21 scores)] before revisions.

**Conclusion:**

Prosthetic mechanical problems, aseptic loosening and infections were primary reasons for revisions after tumor endoprostheses of the knee joint. Although revision surgeries were complicated while still associated with high risk of failure, which remains the remedy strategy for limb salvage and functional recovery in those patients.

## Introduction

With recent advancements in imaging, design of the prostheses, surgical skills and adjuvant chemotherapy for primary malignant bone tumors, limb salvage surgery have become the main strategy for treating those tumors around the knee joint [1]. Various methods, such as autologous bone transplantation, inactive autografting, allografting, tumor endoprostheses, allograft prosthetic composite, and three-dimensional–printed custom-made components have been employed for the reconstruction of the affected limb, of which reconstruction with tumor endoprostheses is most widely used [2–7]. Tumor endoprostheses is advantageous at that it can provide early stability of the affected extremity, allowing a patient to better return social activities [4, 8, 9]. Despite the improvements in the materials used for implants and component designs, potentially serious complications such as prosthetic fracture, infection, mechanical aseptic loosening continue to limit the survival of these endoprosthetic replacements [10]. It was reported that the revision risk of tumor endoprostheses in the knee joint was 17% at 5 years, 33% at 10 years and even more than 50% in 20 years [11]. As a consequence, number of later period revision procedures due to prostheses failure is growing. Similar to the primary endoprostheses, revision surgeries were also associated with a series of complications, such as vascular and nerve injury, prosthetic fracture and loosening, infections, etc. [10, 12]. Even so, revision surgery remains the main solution to preserve the affected extremity and restore functions to date [13]. However, failures, functional outcomes, possible revision strategies, and the survival of these revision implants have not been fully identified.

In a long term, we recruited 144 patients who were diagnosed with malignant tumor around the knee joint, most of these cases received tumor endoprostheses replacement. Synthesize treatments including neoadjuvant and postoperative chemotherapy or targeted therapy have prolonged survival period in those cases, making a revision surgery necessary in a growing number of patients. This study retrospectively analyzed a total of 20 patients admitted by our Bone and Soft Tissue Tumor Center from January 2000 until January 2018 who received revision surgeries due to failure of the primary tumor endoprostheses. Objectives were (1) to summarize different failure types of tumor endoprostheses around the knee joint; and (2) to follow the mid-long-term clinical results and prosthetic survival after revision surgeries.

## Material and methods

A retrospective review was performed of all patients who had undergone a revision surgery due to primary failure of the tumor endoprostheses of knee joint at our Bone and Soft Tissue Tumor Center. Institutional review board approval was obtained before the initiation of the study and all patients consented to the use of their clinical information at the moment of revision. Using a prospectively maintained database, we identified a total of 20 patients with at least one component of prostheses removal during the period of 2000 and 2018. Of whom, 11 were males and 9 were female patients with a mean age of 34.1 years (range, 16 to 66 years) at the time of index revision. The average interval between initial endoprostheses and revision surgery was 101.3 months (range, 32 to 178 months), those patients were followed at least 2 years after revision surgery.

Oncologic diagnoses included 10 osteosarcomas, 5 malignant bone giant cell tumors (MBGCT), 2 Ewing’s sarcomas and 3 chondrosarcomas. 17 of those tumors were located at the distal femur and 3 cases were at proximal tibia. Preoperative examinations included routine X-ray radiographs, computed tomography (CT) scans and magnetic resonance imaging (MRI). Pulmonary CT scan was performed for all patients to exclude any pulmonary metastasis at the time of revision, and positron emission tomography-computed tomography (PET-CT) was conducted if necessary. Indications for a revision surgery would be that the patient was able to tolerate the procedure and had an expectancy of more than 6 months. Patients received routine outpatient follow-up visits at 6 weeks, 3 months, 6 months, 1 year, and every year thereafter postoperatively. Demographic data, operation time, blood loss of before and those after revision were recorded; interval between primary surgery and revision, follow-up time, reasons for revision, and any complications were documented in detail for final analysis. Full length X-ray of both lower extremities was performed to evaluate the mechanical alignment of the limb (center of hip to center of knee to center of ankle), the location and stability of the prostheses. Pulmonary CT scans were conducted to detect any possible metastasis. Local CT scan or systemic isotope bone scan was performed to assess local control of tumor when necessary. Functional outcomes were measured using the 1993 Musculoskeletal Tumor Society (MSTS-93) score [14] preoperatively and 1 year after revision. Reasons for revision were classified according to the system proposed by Henderson et al. [15], this system defines complications as those leading to a revision of the prostheses. They were categorized as mechanical failures: soft-tissue failures (Type 1), aseptic loosening (Type 2), structural failures such as implant fractures, breakage, and periprosthetic fracture (Type 3); and non-mechanical failures: periprosthetic infection (Type 4) and tumor progression (Type 5). Periprosthetic infection was diagnosed through clinical examination, radiographic images, laboratory values and bacterial culture.

When revision surgeries were required, either a one-stage or two-stage procedure was performed. One-stage revision procedure involves removal of the failed prosthetic components and polyethylene parts, debridement of all infected soft tissues; in the meanwhile, new prostheses and other components were implanted to reconstruct the defects. Two-stage revision procedure involves complete removal of all prosthetic components and replaced with antibiotic-loaded bone cement. Systemic antibiotics were administrated to those patients for at least 6 weeks based on the bacterial culture and laboratory values, a revision was performed until white blood cell count, C-reactive protein, and erythrocyte sedimentation rate were normal.

### Statistical analysis

Data was recorded and analyzed using the standard statistical software (SPSS, version22.0, 2013, IBM Corp., Armonk, New York). Categorical variables are presented as numbers and percentages, continuous variables are shown in means and ranges. The Student’s *t* test was used to detect the differences between the preoperative outcomes and those after revision surgeries, including operation time, blood loss and MSTS-93 score. Kaplan-Meier curve analysis was performed to evaluate the prosthetic survival rate. Results were considered statistically significant if a *p* value < 0.05.

## Results

Demographic data are presented in Table 1. Patients were followed at least 2 years with a mean of 64.7 months (range, 27 to 155 months), of whom 11 cases were followed more than 5 years. The mean operation time for primary prosthetic replacements was 163.0 min (range, 130.0 to 190.0 min) and 187.8 min (range, 135.0 to 250.0 min) in the revision surgeries; blood loss was 555.0 ml (range, 300 to 800 ml) and 805.0 ml (range, 400–1500 ml) in the primary surgeries and revisions, respectively, all showing statistically significant differences (*p* < 0.05). Seven of them received preoperative chemotherapy and 11 patients had postoperative chemotherapy; no radiotherapy was performed in those patients. One recurrent osteosarcoma patient died of pulmonary metastasis at 3 years follow-up after revision surgery, and the remaining patients were all alive and free of disease at the most recent follow-up.

**Table 1:** Patients’ demographic data and outcomes

Case	Gender	Age	Pathological diagnosis	Tumor location	Time to revision (m)	Follow-up time (m)	Revision reason	Complications	MSTS-93 score(1year)
1	M	19	Osteosarcoma	DF	45	52	Prosthesis breakage	None	26
2	M	66	Osteosarcoma	DF	126	48	Prosthesis breakage	None	27
3	M	16	Osteosarcoma	DF	32	60	Prosthesis bending	Wound complications	23
4	M	57	Osteosarcoma	DF	178	72	Proximal prosthesis break out	None	23
5	M	42	Osteosarcoma	DF	152	45	Screw breakage, LLD, pain	None	24
6	F	62	Chondrosarcoma	DF	162	72	Periprosthetic fracture	None	23
7	F	28	MBGCT	DF	96	70	MRSA infection	Infection without control, amputation	excluded
8	F	26	Osteosarcoma	DF	121	77	Prosthesis breakage	Infection, spacer-second revision	15
9	F	59	MBGCT	DF	123	155	Prosthesis wear and loosen	Prosthesis loosen, second revision	18
10	M	23	MBGCT	PT	132	77	Prosthesis loosen	Infection	16
11	F	45	Osteosarcoma	PT	106	55	Prosthesis loosen	None	26
12	M	22	Osteosarcoma	DF	61	66	Prosthesis loosen	Wound complications	18
13	M	23	Osteosarcoma	DF	66	65	Prosthesis breakage	None	23
14	F	44	MBGCT	DF	76	48	Prosthesis breakage	None	21
15	F	18	Chondrosarcoma	DF	145	27	Infection	None	18
16	M	16	Osteosarcoma	DF	38	36	Tumor recurrence	Pulmonary metastasis, die	16
17	F	23	MBGCT	PT	93	44	Infection	None	16
18	M	18	Ewing sarcoma	DF	84	47	Prosthesis breakage	None	21
19	M	20	Ewing sarcoma	DF	155	85	Prosthesis loosen	None	20
20	F	55	Chondrosarcoma	DF	35	93	Prosthesis breakage	None	24

*LLD* leg length discrepancy, *MBGCT* malignant bone giant cell tumor, *DF* distal femur, *PT* proximal tibia, *MRSA* methicillin-resistant Staphylococcus aureus

**Fig. 1:**
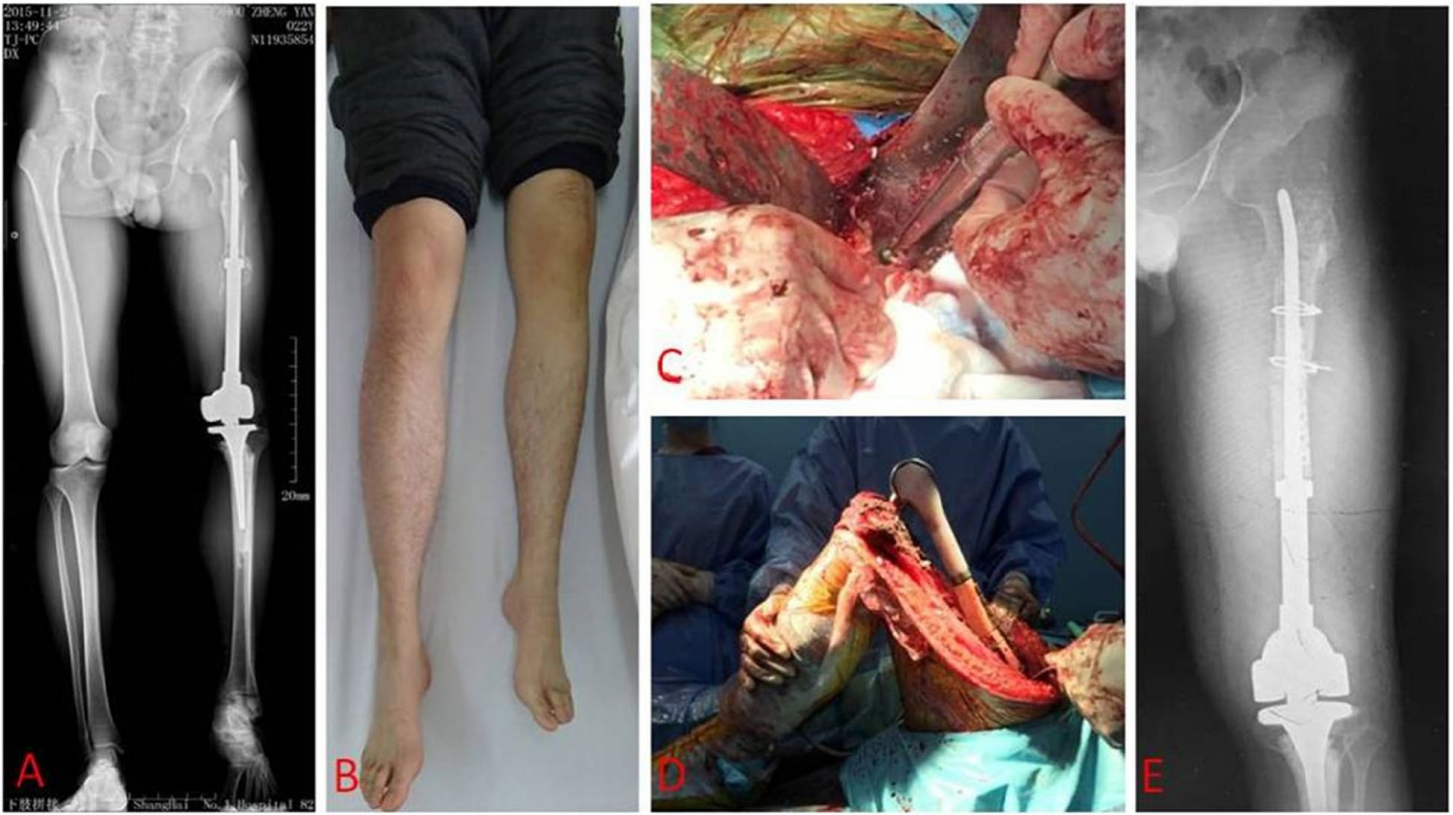
A 57-year-old male osteosarcoma patient, 14 years after initial endoprosthetic replacement. **A** Preoperative X-ray showed proximal femur prosthetic component loosening and breakout. **B** The shortened affected extremity, leg length discrepancy (LLD). **C** Allograft segment, two cables, plate, and screws were used to reconstruct and fix the proximal prostheses. **D** Proximal component was rebuilt. **E**, postoperative 1-year X-ray showed prostheses and other components in position

**Fig. 2:**
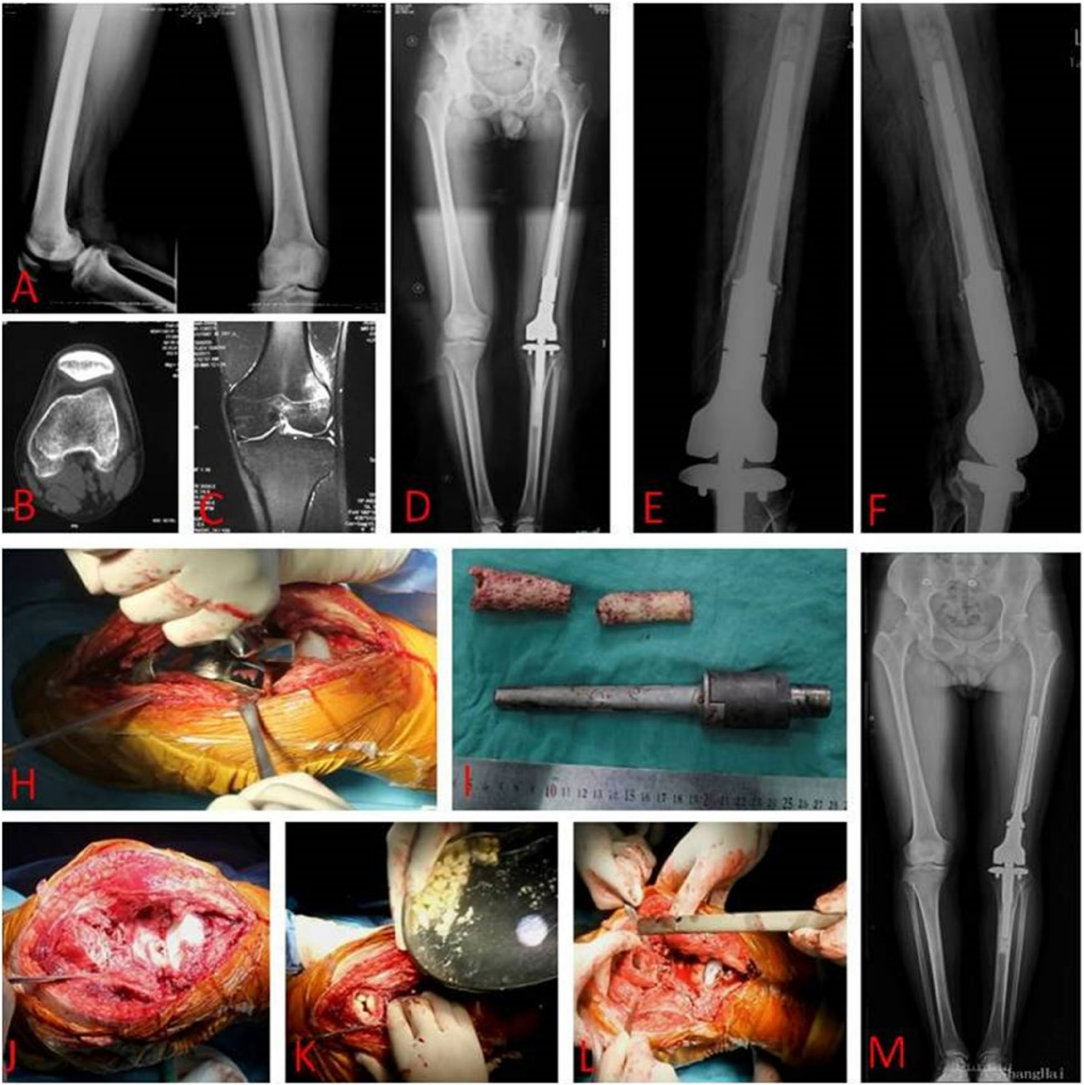
A 22-year-old osteosarcoma male patient, underwent a revision surgery because of the prostheses loosening. **A**–**C** Preoperative X-ray, CT-scan, and MRI image showed a lesion at the distal femur. **D** Postoperative 1-year X-ray showed the prosthetic components at position. **E**, **F** Postoperative 3-year X-ray showed a prostheses loosening at the femur site. **H**, **I**, Prosthetic components at the femur site were removed. **J**, **K** The residual cement was removed and followed with allograft implanted. **L** A biological prosthetic component was implanted. **M** Postoperative full length film of double lower extremities showed the prostheses at position

Types of failures were summarized in Table 2 and two revision cases were presented as Figs. 1 and 2. Failed primary endoprostheses requiring revision included 5 cases of Type 2 failures (20.0%), 11 cases of Type 3 failures (55.0%), 3 cases of Type 4 failures (25.0%), and 1 case of Type 5 failure (5.0%); and there were no cases of Type 1 failures in this cohort. Type 2 failure (aseptic loosening) occurred at a mean of 115.4 months (range, 61 to 155 months). A total of 5 patients experienced this failure, and all received a one-stage revision procedure. Those patients were followed at a mean of 87.6 months (range, 55 to 155 months), of whom, one case experienced a second aseptic loosening 6 years after the initial revision and received a second revision; one case had postoperative early infection after revision and healed by debridement and antibiotics administration; another case experienced wound complication and treated with numbers of dressing changes. Other two cases were without any accident in the latest follow-up.

**Table 2:** Type of failures

Type of failures	Type 1 (soft tissue failure)	Type 2 (aseptic loosening)	Type 3 (structural failure)	Type4 (periprosthetic infection)	Type 5 (tumor progression)
Primary endoprostheses (*n* = 20)	None	5(25.0%)	11(55.0%)	3(15.0%)	1(5.0%)
Revisions (*n* = 7)	2(28.6%)	1(14.3%)	None	3(42.9%)	1(14.3%)

Type 3 failures (structural failures) accounted for 55.0% (11/20) of all the revisions, and they happened at a mean of 97.9 months (range, 32 to 178 months) after the primary endoprostheses. Those patients were followed at a mean of 61.7 months (range, 45 to 93 months) after revisions, one patient experienced deep infection in the early postoperative period after the revision procedure and received a two-stage revision surgery; another case had wound complications and treated with debridement and antibiotics therapy.

Type 4 failures (periprosthetic infection) occurred in 3 patients at a mean time of 111.3 months (range, 93 to 145 months) and those patients were followed 47 months (range, 27 to 70 months) after the revisions. All the 3 cases received two-stage revision procedures with prostheses removal and antibiotic-loaded bone cement in occupation initially. One patient was cultured with Methicillin-resistant Staphylococcus aureus (MRSA); although a two-stage revision was performed, the patient experienced persistent infections after revision and received amputation procedure in the end. The other two cases were disease-free at the latest follow-up period.

Type 5 failures (tumor progression) occurred in one case in this cohort, and this patient experienced tumor recurrence 38 months after the primary endoprostheses and received a second tumor resection with prostheses reconstruction. In the following, the patient was diagnosed with pulmonary metastasis receiving multiple chemotherapies and died at 3 years after the revision surgery.

Kaplan-Meier survival curve was plotted, which indicated that 5-year survival rate of the initial prostheses in the revision cohort was 75% and 10-year survival rate was 40%. Functional MSTS-93 scores were obtained in all the cases before and 1 year after revision procedures. The average overall score was 17.2 (range, 13 to 21 scores) before revision and 20.9 (range, 15 to 27 scores) 1 year after revision, significant difference was detected in between (*p* < 0.001).

## Discussion

The introduction of neoadjuvant and adjuvant chemotherapy allowed resection and reconstruction instead of amputation as surgical treatment in most cases of bone sarcomas [16–18]. Segmental metallic endoprostheses have been more frequently used and play an increasingly important role in limb reconstruction after resection of long bones around the knee joint [15]. However, failures such as infection, tumor progression, aseptic lessening, prosthetic breakage, and periprothestic fracture may occur during the follow-up period [4, 10, 19, 20]. It was reported that failures of the endoprosthetic reconstruction range from 17 to 75% at 5 to 15 years [11, 21, 22]. Therefore, it will be of clinical significance to summarize the failures, clinical outcomes, and prosthetic survival of the tumor endoprosthses replacement around the knee joint.

Prosthetic mechanical problems after tumor endoprostheses reconstruction were the primary reasons for a revision surgery, followed by aseptic loosening and infection. Soft tissue failure did not occur in our cohort, and tumor progression was detected in one case, as prosthetic revision was rarely necessary in most cases of soft tissue failure and tumor progression. Overall, the reports in the literatures had similar results to our findings [15, 23, 24]. In Henderson’s report [15], the most common mode of failure was infection, followed by aseptic loosening and structural failures, soft tissue problem and tumor progression accounted less, whereas in their cohort different kinds of endoprostheses were included, such as proximal humeral replacements, total humeral replacement, distal humeral replacements, proximal femoral replacements, total femoral replacement, distal femoral replacement, combined distal femoral-proximal tibial replacements, and proximal tibial replacements. Biau and colleagues [23] reported a total of 91 cases of tumor endoprotheses around the knee joint, 36 received revision surgeries, of which 23 were mechanical problems, 7 were infection, and 6 were tumor recurrence. Wirganowicz et al. [24] reported in their 64 failure cases that aseptic loosening and mechanical failure accounted for most of the failures and they were revised successfully; a total of 9 patients experienced tumor recurrence, of whom 8 received amputation surgery.

The infection rate of distal femur tumor endoprostheses was reported in the literature as 5.5% and range from 3.6 to 40% in the proximal tibia [15, 25, 26]. Theoretically, the risk of infection in the proximal tibia had increased because of the relative lack of wound coverage and unreliable extensor mechanism reconstruction [27]. In our series 2 infection occurred in the distal femur and 1 was in the proximal tibia, a two-stage revision was performed with spacer implanted initially and new prostheses were implanted after infection control. Infections after revision surgeries remain a vital problem in those patients, and three cases experienced a second infection after revision surgery, a MRSA infection patient had continuous infection and received amputation in the end, another one received a two-stage revision and the last one healed by debridement. Most literatures suggested a two-stage revision instead of one-stage for those tumor endoprostheses replacements [25, 28, 29]. Although a literature reported the successful rate of early stage in a one-stage revision is high, it continues to decline to 14% in the long follow-up period [30]. The success rate in a two-stage revision for those infections in the tumor endoprostheses replacements was reported as high as 74% [25]. We conducted 4 cases of two-stage infection revisions, one case had infection uncontrolled and received amputation.

Our results showed that overall 13.9% of the patients required a revision of their initial prostheses, this rate was lower than those reported in literatures [11, 15, 31] as some failures failed to have the opportunity to revise in our cohort. Studies published reported that a survival rate of up to 87% at 3 years and 67 to 88% at 5 years, this decreased to 48 to 65% at 10 years and very limited data was available at 20 years [32–34]. In Wirganowicz’s report [24], the 5-year survival rate of the prostheses in their cohort was 81%, and another report showed a 5-year survival rate of the prostheses in their revision group of 79% and a 10-year survival rate of 65% [33]. In our cohort, the 5-year survival rate of initial prostheses in the revised cohort was 75%, and this decreased to 40% at 10-years, which was co-insistent with reports in the literatures. Tumor endoprostheses replacement around the knee joint mostly achieved better functions of the extremities. In Kawai’s report [33], range of motion of the knee joint after tumor endoprostheses reached to approximate 90° and MSTS-93 scores were 80% at an average. However, revised tumor endoprostheses experienced inferior functional results. Shin et al. [35] reported 19 revised cases of tumor endoprostheses replacements, a 10-year MSTS-93 score was only 57%. Reasons for revision may introduce major effect to postoperative functional outcomes, patients revised of mechanical failure mostly achieve better functions, whereas infection cases always had inferior functional recovery [36]. In our cohort, the MSTS-93 scores were significantly improved compared with the values before revision, which indicated a better functional recovery in these revision cases, especially for those patients with mechanical problems and aseptic loosening.

Several limitations of the present study should be addressed as follows. First, it is a retrospective, non-randomized case series with a small sample size which may have introduced potential selection bias. Second, the tumors of the included patients in the study are heterogeneous regarding biological behavior and stages, and the intervention of neoadjuvant and adjuvant chemotherapy may have affected the oncologic prognosis and results. Third, although comparisons were conducted in different type of prosthetic failures, primary and revision surgeries, this study lacked a true control group. Whereas, we opted to include all patients who received prosthetic revisions after limb salvage surgery in a long period to address failure types, functional outcomes, and prosthetic survival in revision tumor endoprostheses of the knee joint, even with limitations, our results may be of meaningful.

## Conclusion

Tumor endoprostheses replacements of knee joint are associated with high rate of failures with mechanical problems, aseptic loosening, and infection as the primary reasons. Even revision surgery is challenging as well as accompanied with high rate of complications, it is still the main solution for those patients to preserve the affect limb and restore functions.

## Data Availability

The dataset supporting the conclusions of this article is available on request—please contact the corresponding author.

## Abbreviations

MBGCT: Malignant bone giant cell tumor
MSTS-93: Musculoskeletal Tumor Society 1993
